# Quantity of IgG response to SARS-CoV-2 spike glycoprotein predicts pulmonary recovery from COVID-19

**DOI:** 10.1038/s41598-022-07489-6

**Published:** 2022-03-07

**Authors:** Manfred Nairz, Sabina Sahanic, Alex Pizzini, Anna Böhm, Piotr Tymoszuk, Anna-Maria Mitterstiller, Laura von Raffay, Philipp Grubwieser, Rosa Bellmann-Weiler, Sabine Koppelstätter, Andrea Schroll, David Haschka, Martina Zimmermann, Silvia Blunder, Kristina Trattnig, Helene Naschberger, Werner Klotz, Igor Theurl, Verena Petzer, Clemens Gehrer, John E. Mindur, Anna Luger, Christoph Schwabl, Gerlig Widmann, Günter Weiss, Judith Löffler-Ragg, Ivan Tancevski, Thomas Sonnweber

**Affiliations:** 1 Department of Internal Medicine II, Medical University of Innsbruck, Innsbruck, Austria; 2 Department of Internal Medicine V, Medical University of Innsbruck, Innsbruck, Austria; 3 Repertoire Immune Medicines, Cambridge, MA USA; 4 Department of Radiology, Medical University of Innsbruck, Innsbruck, Austria; 5 Christian Doppler Laboratory for Iron Metabolism and Anemia Research, Medical University of Innsbruck, Innsbruck, Austria

**Keywords:** Viral infection, Viral infection

## Abstract

The CovILD study is a prospective, multicenter, observational cohort study to systematically follow up patients after coronavirus disease-2019 (COVID-19). We extensively evaluated 145 COVID-19 patients at 3 follow-up visits scheduled for 60, 100, and 180 days after initial confirmed diagnosis based on typical symptoms and a positive reverse transcription-polymerase chain reaction (RT-PCR) for severe acute respiratory syndrome coronavirus-2 (SARS-CoV-2). We employed comprehensive pulmonary function and laboratory tests, including serum concentrations of IgG against the viral spike (S) glycoprotein, and compared the results to clinical data and chest computed tomography (CT). We found that at the 60 day follow-up, 131 of 145 (90.3%) participants displayed S-specific serum IgG levels above the cut-off threshold. Notably, the highly elevated IgG levels against S glycoprotein positively correlated with biomarkers of immune activation and negatively correlated with pulmonary function and the extent of pulmonary CT abnormalities. Based on the association between serum S glycoprotein-specific IgG and clinical outcome, we generated an S-specific IgG-based recovery score that, when applied in the early convalescent phase, accurately predicted delayed pulmonary recovery after COVID-19. Therefore, we propose that S-specific IgG levels serve as a useful immunological surrogate marker for identifying at-risk individuals with persistent pulmonary injury who may require intensive follow-up care after COVID-19.

## Introduction

Severe acute respiratory syndrome coronavirus 2 (SARS-CoV-2) causes a spectrum of subclinical and clinical manifestations ranging from asymptomatic infection to fatal respiratory and systemic disease^1–3^. To date, a plethora of clinical studies were initiated to elucidate the underlying pathophysiology of coronavirus disease 2019 (COVID-19). However, we still lack a comprehensive understanding of the immunopathology of COVID-19 and of the effects the immune system exerts on recovery from acute disease.

Host–pathogen interplay occurring in the acute phase of SARS-CoV-2 infection is a major dictating factor in the disease course of COVID-19. This interplay is shaped by local and systemic immune responses against SARS-CoV-2, the spreading of virus in the respiratory tract, and by immune- and pathogen-mediated effects on pulmonary and other systems of the host organism^4,5^. The immune response against respiratory viruses, including SARS-CoV-2, involves rapid innate and subsequent adaptive immune mechanisms^6–9^. To assess the course of COVID-19 in hospitalized patients, innate immune responses can be monitored in the peripheral blood by measuring concentrations of the master inducer of the acute-phase response, interleukin (IL)-6, and its down-stream effectors, such as C-reactive protein (CRP) and ferritin. Adaptive immune mechanisms, on the other hand, are largely mediated by B and T lymphocytes, which give rise to antibody and cytokine-mediated responses^10,11^. Whereas pathogen-specific T cell responses are laborious to assess in a routine diagnostic setting, B cell-mediated immunity can be more easily tracked by the quantitative measurement of antibody levels^12^. In this swiftly evolving field, enzyme-linked immunosorbent assays (ELISA) and automated chemiluminescence immunoassays (CLIA) have emerged as the most widely available tests to assess antibody levels. The spike (S) glycoprotein and its receptor-binding domain (RBD) as well as the nucleocapsid (N) protein are common targets of the B cell response and are thus frequently used as antigens to assess humoral immune responses via specific antibody detection^13–17^.

In respiratory infections, three antibody isotypes, IgM, IgG, and IgA, are primarily produced to mediate prompt, long-lasting, and mucosal immunity, respectively^18–20^. In response to SARS-CoV-2 infection, these 3 isotypes emerge early and nearly simultaneously in the serum^21^. Secretory IgA confers strong protection against the virus as assessed by neutralization studies^22,23^, whereas IgG persists in the serum for months, suggesting relatively long-lasting protection^24–30^. Failure to generate sufficient IgG antibodies is linked to reduced survival^31^. Other studies, however, found a positive^17,32–34^ or unclear association^35^ between serum concentrations of IgG antibodies against SARS-CoV-2 and COVID-19 disease severity. These findings are further complicated by the fact that some individuals carry pre-existing antibodies that can neutralize SARS-CoV-2^36^, and antibody responses are age-dependent^37^. Hence, the effects of SARS-CoV-2-specific IgG concentrations on the clinical course of COVID-19 remain controversial.

The work presented herein aimed to investigate yet another aspect of COVID-19; more specifically, we assessed whether the quantity of IgG response is associated with lung damage and able to predict pulmonary recovery from symptomatic SARS-CoV-2 infection.

## Results

### Clinical and demographic patient characteristics

A total of 145 symptomatic adults who previously suffered from mild to critical COVID-19 were included in the study and attended 3 follow-up appointments scheduled for 60, 100, and 180 days after the initial confirmed diagnosis of COVID-19. The mean age of study participants was 57 years (SD ± 14 years) and the majority were male (57%). Clinical and demographic characteristics of the CovILD cohort are detailed in Table 1.

**Table 1:** Demographic and clinical characteristics of COVID-19 patients of the CovILD study.

	N = 145
**Characteristics**	
Mean age—year (SD)	57 (14)
Female sex—no. (%)	63 (43)
Mean body mass index—kg/m^2^ (SD)*	26 (5)
Smoking history—no. (%)	57 (39)
Comorbidities—no. (%)	
None	33 (23)
Cardiovascular disease	58 (40)
Hypertension	44 (30)
Pulmonary disease	27 (19)
COPD	8 (6)
Asthma	10 (7)
Metabolic disease	63 (43)
Chronic kidney disease	10 (7)
Chronic liver disease	8 (6)
Malignancy	17 (12)
Immunodeficiency^‡^	10 (7)
Hospitalized—no. (%)	109 (75)
In-hospital treatment^§^	
Oxygen supply—no. (%)	72 (66)
Non-invasive ventilation—no. (%)	3 (3)
Invasive ventilation—no. (%)	29 (27)

*The body-mass index is the weight kilograms divided by the square of the height in meters. ^‡^Due to disease or ongoing immunosuppressive treatment: renal transplantation (N = 1), psoriasis vulgaris (N = 1), Morbus Hashimoto (N = 1), leukaemia (N = 1), lymphoma (N = 1), gout (N = 1), myasthenia gravis (N = 1), polyarthritis (N = 3); ^§^All patients needing non-invasive or invasive ventilation were supplied with oxygen before ICU admission, relative numbers depict the treatment of in-hospital patients.

Disease severity ranged from mild to critical according to medical treatment need. Specifically, 36 patients (25%) developed mild disease (outpatient treatment), 37 patients (26%) developed moderate disease (inpatient treatment without respiratory support), 40 patients (28%) developed severe disease (inpatient treatment with respiratory support), and 32 patients (22%) developed critical disease and required mechanical ventilation in the intensive care unit (ICU).

### S-specific IgG levels correlate with the severity of acute COVID-19

**Figure 1:**
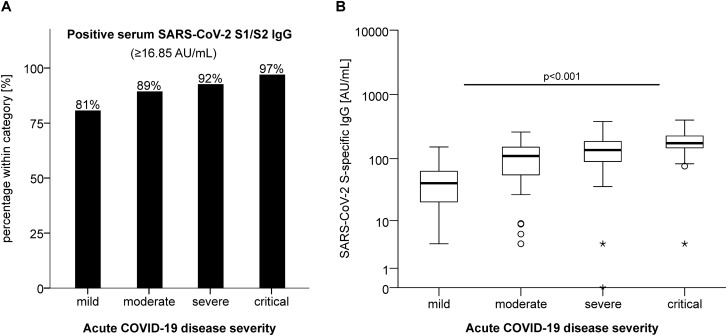
Qualitative and quantitative results for S-specific IgG correlate with the clinical severity of COVID-19. Patients were categorized according to clinical severity of acute COVID-19 (N_mild_ = 36, N_moderate_ = 37, N_severe_ = 40, N_critical_ = 32). For each clinical category of disease severity, the relative abundance of patients (**A**) who mounted a substantial IgG response against the S glycoprotein above the cut-off threshold is depicted. The SARS-CoV-2 IgG concentrations were quantified (**B**) at the 60 days follow-up according to acute disease severity categories. p-values were calculated with the Kruskal–Wallis test.

First, we asked whether serum concentrations of IgG specific for the S glycoprotein of SARS-CoV-2 are associated with the disease course. To this end, we used a certified *in-vitro* diagnostic (IVD) chemiluminescence immunoassay (CLIA) and a validated cut-off threshold of 16.85 AU/mL to classify study participants, all of whom had been symptomatic and diagnosed prior by reverse transcription-polymerase chain reaction (RT-PCR) as negative or positive for S-specific IgG. According to this qualitative classification, we found that 81% of patients with mild COVID-19 (N = 29), 89% with moderate disease (N = 33), 92% with severe disease (N = 37), and 97% with critical disease (N = 31) produced substantial amounts of S-specific IgG (Fig. 1A). Notably, the only patient with critical COVID-19 who did not mount a detectable antibody response against S glycoprotein had received rituximab, a therapeutic CD20-specific monoclonal antibody, eight weeks before SARS-CoV-2 infection. In contrast, none of the other study participants had undergone any treatment known to directly affect antibody production or half-life (e.g. B cell depletion or plasmapheresis).

Second, we compared the quantity of S-specific IgG measured at the 60-day follow-up to the classified severity of acute COVID-19 disease from which the study participants were recovering. We observed that levels of S-specific IgG and severity of COVID-19 were positively correlated (Fig. 1B). Therefore, outpatients with mild disease displayed the lowest antibody levels and ICU patients with critical disease displayed the highest antibody levels at the 60-day follow-up.

**Figure 2:**
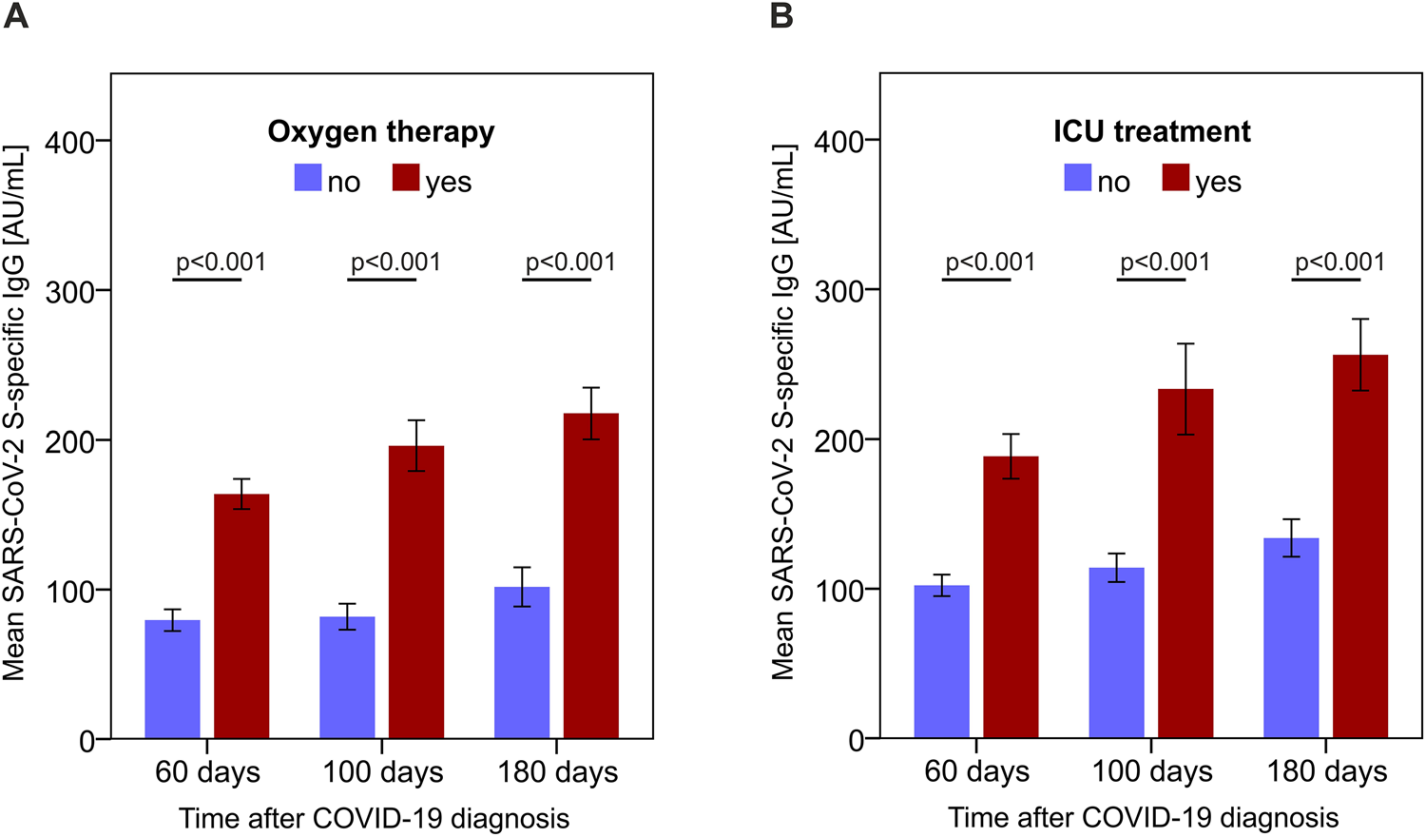
S-specific IgG levels correlate with supplemental O_2_ requirement and intensive care during acute COVID-19. S-specific IgG serum concentrations are reported according to need for oxygen supply (N_Y/N_ = 72/73) or ICU treatment (N_Y/N_ = 32/113) during acute COVID-19. P-values were calculated with the Mann–Whitney-*U* test. N_60days_ = 145; N_100days_ = 135; N_180days_ = 118.

Third, we analyzed whether the correlation between S-specific IgG levels and clinical disease course is linked to the exclusive requirement for O_2_ therapy or intensive care, respectively, and if the differences in quantity of S-specific IgG at the 60-day follow-up were still present at the 100- and 180- day reevaluation. We found that patients requiring O_2_ therapy had significantly higher S-specific IgG levels at all time-points throughout the observation period in comparison to patients who never required supplemental O_2_ (Fig. 2A). Similarly, patients admitted to the ICU for critical acute COVID-19 disease displayed significantly higher S-specific IgG levels after 60, 100, and 180 days compared to patients who did not require intensive care (Fig. 2B). Taken together, these data indicate the S glycoprotein-specific IgG response serves as a reliable clinical correlate for acute COVID-19 disease severity, tracking with the degree of patient supportive care.

### Correlations of S-specific IgG levels with other biomarkers of COVID-19

**Figure 3:**
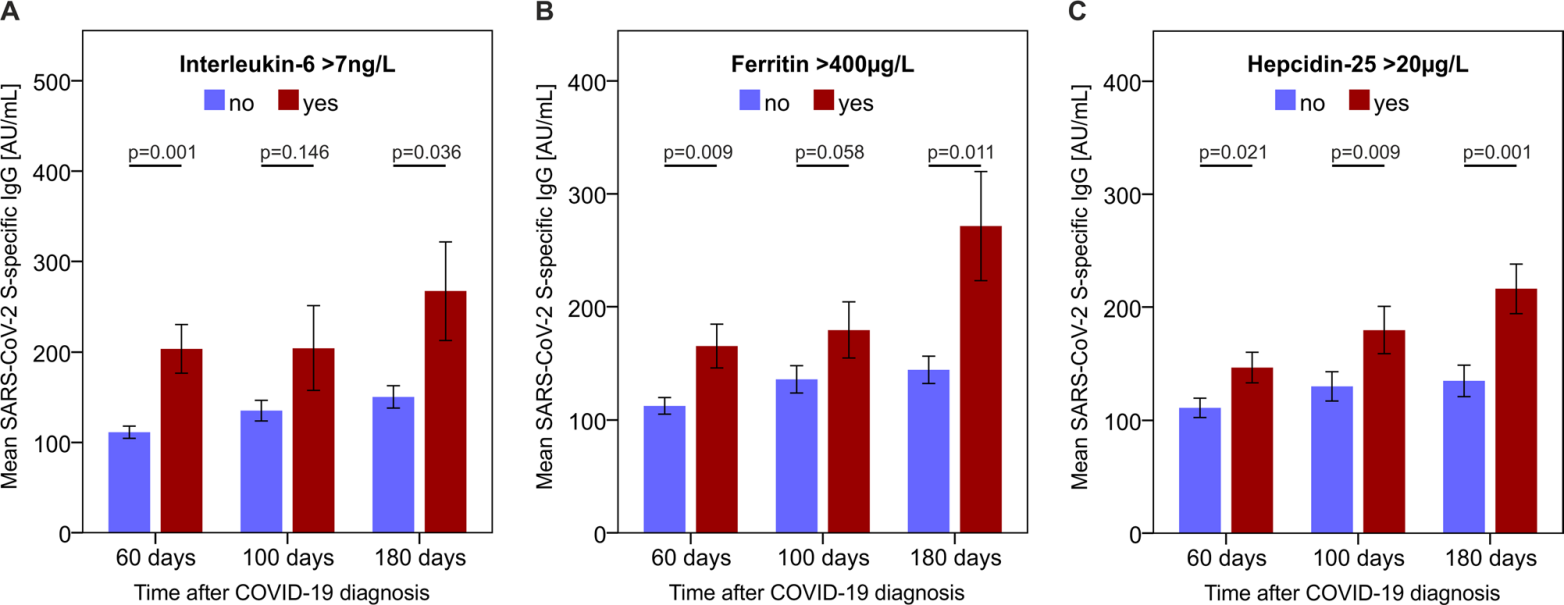
S-specific IgG is associated with elevated serum IL-6 levels, ferritin, and hepcidin-25. Patients with persistingly elevated IL-6 (cut-off: 7 ng/L) (**A**), ferritin (> 400 µg/L) (**B**), and hepcidin-25 (> 20 µg/L) (**C**) demonstrate significantly higher S-specific IgG concentrations compared to individuals without elevated IL-6 at follow-up. 12%, 6%, and 4% of patients demonstrated increased IL-6 levels at the 60-day, 100-day, and 180-day follow-up, respectively. N_60days_ = 145; N_100days_ = 135; N_180days_ = 118.

Next, we extended our analyses to evaluate known biomarkers associated with COVID-19 disease severity. We saw that at all follow-up visits, patients with persistently elevated IL-6 levels (cut-off: 7 ng/L), as well as serum ferritin (cut-off: 400 µg/L) and hepcidin-25 (cut-off: 20 µg/L), showed higher S-specific IgG concentrations (Fig. 3). In line with known biomarkers of acute COVID-19 disease severity, these results further highlight the association between the S glycoprotein-specific IgG response and COVID-19 severity.

### Levels of S-specific IgG are associated with persistently impaired pulmonary function

**Figure 4:**
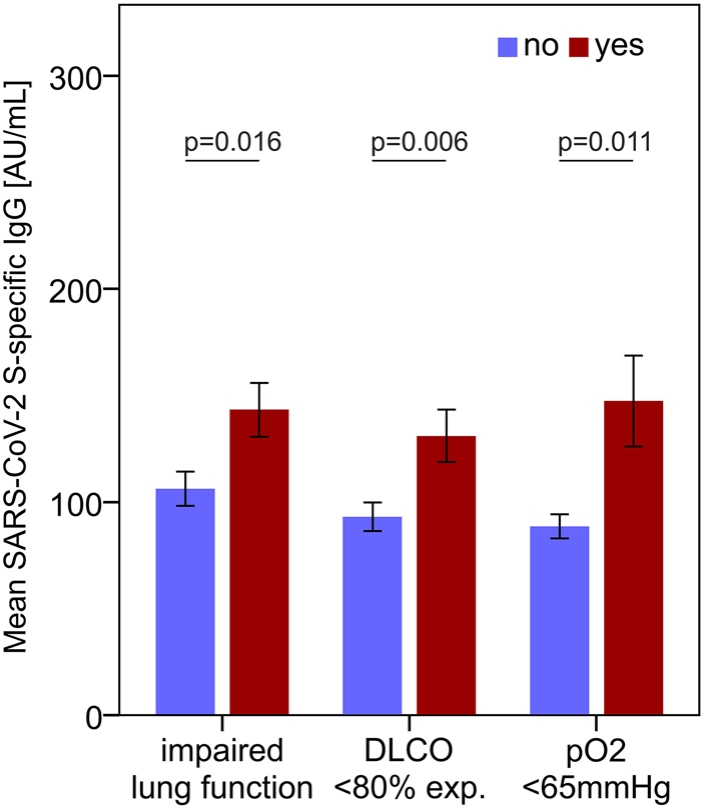
S-specific IgG is related to pulmonary function and hypoxia. S-specific IgG levels are increased in patients with impaired lung function and hypoxia at the 60-day follow-up. Serum S-specific IgG concentrations in patients with any impairment of lung function (including reduced FEV1, FVC, TLC, or DLCO), reduction of diffusion capacity for carbon monoxide (DLCO), or significant hypoxia (defined by an arterial pO_2_ < 65 mmHg) are shown. P-values were calculated with the Mann–Whitney U test. N_60days_ = 145; N_100days_ = 135; N_180days_ = 118.

Given the relation between serum S-specific IgG concentrations and COVID-19 severity, we then asked if S-specific SARS-CoV-2 IgG also correlated with pulmonary recovery. Thus, we searched for a correlation between concentrations of S-specific IgG and pulmonary function based on functional lung assessments performed by body plethysmography. Notably, we found that patients with impaired lung function, including measurements of forced expiratory volume in 1 s (FEV1; cut-off 80% of the calculated normal value), forced vital capacity (FVC; cut-off 80% of normal), FEV1/FVC (cut-off 70%) or total lung capacity (TLC; cut-off 80% of normal), as well as a reduction of the diffusion capacity for carbon monoxide (DLCO) below normal levels (cut-off 80% of the normal level calculated from sex, age, and height) and hypoxia (arterial pO_2_; cut-off 65 mmHg), were related to increased S-specific IgG levels at the 60-day follow-up (Fig. 4).

### Levels of S-specific IgG are associated with persistent abnormalities in pulmonary computed tomography

**Figure 5:**
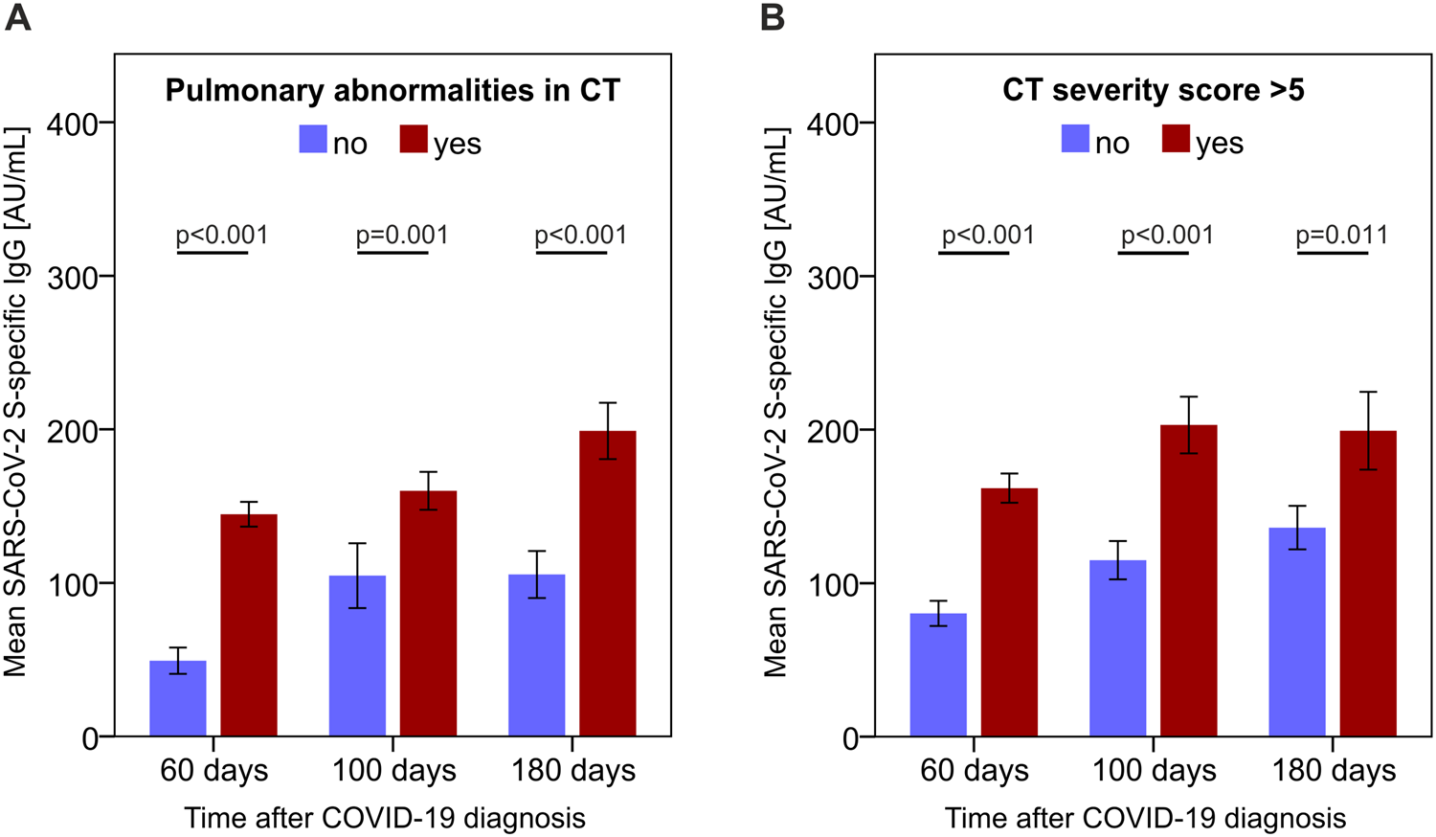
S-specific IgG levels correlate with pulmonary CT abnormalities. Structural pulmonary abnormalities were assessed with computed tomography (CT) and S-specific IgG serum concentrations according to (**A**) the presence of pulmonary CT abnormalities and (**B**) the presence of more pronounced CT abnormalities are shown. P-values were calculated with the Mann–Whitney U test. N_60days_ = 145 (N_pulmonary findings_ = 113, N_CT severity score >5_ = 72); N_100days_ = 135 (N_pulmonary findings_ = 82, N_CT severity score >5_ = 40); N_180days_ = 118 (N_pulmonary findings_ = 52, N_CT severity score >5_ = 23).

Next, we looked for possible correlations between S-specific IgG levels and structural pulmonary findings based on computed tomography (CT) assessments. We found that patients with pulmonary CT abnormalities at follow-up had higher levels of S-specific IgG concentrations in comparison to individuals who either lacked CT abnormalities or resolved such abnormalities by the follow-up (Fig. 5). We observed a similar correlative outcome when we stratified patients according to the severity of pulmonary CT abnormalities and compared S-specific IgG levels in individuals with no or very mild pulmonary CT abnormalities (severity score of 0–5 points) to those with more prominent CT lung abnormalities (CT severity score > 5 points). Importantly, pulmonary CT abnormalities could be linked to S-specific IgG levels at each of the 3 follow-up visits (Fig. 5).

### Assessment of S-specific IgG is useful to generate a pulmonary recovery score associated with lung recovery following COVID-19

**Figure 6:**
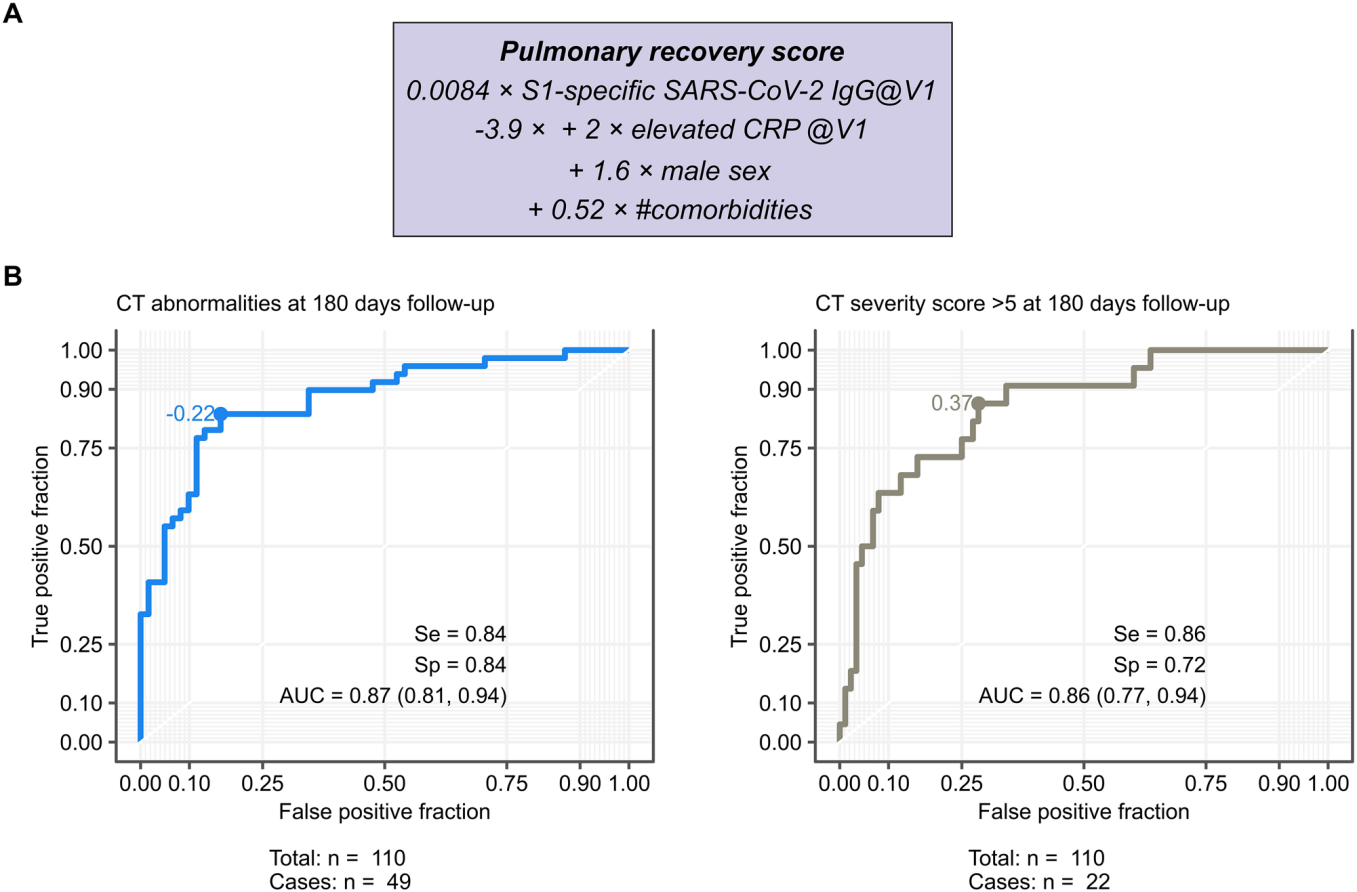
A pulmonary model incorporating early S-specific IgG measurement predicts pulmonary recovery at long-term follow-up. (**A**) We used S-specific IgG measurements acquired at the 60 days follow-up after RT-PCR-based diagnosis of COVID-19 to generate a score predicting structural lung recovery. (**B**) ROC analysis of the score used to predict the presence of pulmonary CT abnormalities and CT abnormalities with a CT severity score > 5 at 180 days follow-up are shown.

Since S-specific IgG levels associated with impaired functional and structural pulmonary findings, we finally tested if S-specific serum IgG combined with other demographic and laboratory markers recorded at the 60-day follow-up could predict the risk of persistent lung pathologies in COVID-19 convalescents. We initially investigated the link between lung abnormality persistence by CT at the 180-day follow-up and known risk factors for COVID-19 severity, including male sex, the presence of comorbidities (as listed in Table 1), and serum levels of CRP and S-specific IgG determined at the 60-day follow-up. As shown in the Table 2, our univariate logistic model analysis uncovered a significant link between the investigated risk factors and structural lung recovery. Importantly, the tested parameters remained significant when combined in a multivariate fashion, suggesting an additive, non-colinear prediction value of non-recovering lung pathology for sex, comorbidity count, CRP levels, and S-specific IgG (Table 2). Thus, we combined various risk parameters to develop a pulmonary recovery score based on the linear coefficients of the multivariate logistic model (Fig. 6). Notably, the resulting lung recovery score demonstrated prediction accuracies exceeding 87% and a sensitivity over 84%.

**Table 2:** Results of univariate and multivariate risk modeling for parameters of the biosignature score.

Parameter	Univariate logistic regression		Multivariate logistic regression			
	CT abnormalities OR (95%CI)	CT severity score > 5 OR (95%CI)	CT abnormalities		CT severity score	
			OR (95%CI)	Δ deviance	OR (95%CI)	Δ deviance
Elevated CRP @V1	5.7 (1.9, 21) p = 0.0041	6.1 (2, 19) p = 0.0046	7.2 (1.7, 40) p = 0.017	− 7.2 p = 0.01	6.6 (1.7, 29) p = 0.019	− 7.5 p = 0.017
Sex male	3.9 (1.8, 8.8) p = 0.0014	3.7 (1.3, 12) p = 0.017	5 (1.8, 16) p = 0.0059	− 9.5 p = 0.0041	4.9 (1.4, 23) p = 0.026	− 6.2 p = 0.017
# comorbidities	1.8 (1.4, 2.4) p = 3.2e−05	1.4 (1.1, 1.8) p = 0.0046	1.7 (1.3, 2.3) p = 0.00029	− 19 p = 4.2e−05	1.3 (1, 1.8) p = 0.026	− 5.3 p = 0.022
SARS-CoV-2 anti-S-IgG @V1	1 (1, 1) p = 0.00049	1 (1, 1) p = 0.0051	1 (1, 1) p = 0.021	-5.7 p = 0.017	1 (1, 1) p = 0.026	− 6.2 p = 0.017

Correlations with presence of CT abnormalities at the 180 day follow-up were assessed by logistic regression. Significance of model terms was determined by Wald z-test. For the multivariate model, significance of particular terms was additionally assessed by likelihood-ratio tests (LRT, Chi^2^ test for Δ deviance ≠ 0). p-values were corrected for multiple comparisons with the Benjamini–Hochberg method.

## Discussion

SARS-CoV-2 causes a spectrum of manifestations that range from subclinical to severe and life-threatening infections. In patients with symptomatic disease, clinical complaints are diverse and include mild dry cough as well as loss of smell and taste, but also multiorgan dysfunction with hyperinflammation, acute respiratory distress syndrome, cardiovascular dysfunction, and coagulopathy^38–40^. A growing number of studies have shown that plasma biomarkers such as IL-6, ferritin, and D-dimers are useful for identifying patients at increased risk for such clinical complications, severe disease, and mortality^38,41–44^. However, we still lack parameters that can accurately predict delayed recovery in survivors of COVID-19 after hospital discharge^45^.

In the prospective COVID-19 follow-up study reported herein, we found that the magnitude of antibody response against the S glycoprotein of the causative infectious agent, SARS-CoV-2, is linked to pulmonary recovery based on laboratory evaluation, pulmonary function testing, and CT morphology assessments. Specifically, high concentrations of S-specific IgG, measured approximately 60 days after disease onset in outpatients followed up for COVID-19, identified individuals with abnormalities in pulmonary function and imaging studies persisting until 180 days after acute disease. Moreover, our data suggest that the levels of SARS-CoV-2 S-specific IgG, in conjunction with patient demographics and laboratory signs of inflammation, may be used to create a valuable recovery score for patient assessment in the early convalescent phase of the disease. This score allowed us to predict delayed pulmonary recovery with high accuracy, but it remains to be validated in independent patient cohorts.

In severe acute infections, a strong and dysregulated immune response characterized by a ‘cytokine storm’ can lead to multiorgan dysfunction^46,47^. This has been proposed for both the 1918 H1N1 influenza pandemic^48,49^ and the ongoing SARS-CoV-2 pandemic^50^. However, other evidence suggests that cytokine production may rather be impaired during SARS-CoV-2 infection and that insufficient activation of innate and adaptive immunity may contribute to severe and fatal COVID-19^51,52^. Moreover, the inability to mount an appropriate IgG response against SARS-CoV-2 is linked to reduced survival. This association is best described for groups of patients who received the B cell-depleting monoclonal antibody, rituximab, within 6 months before contracting COVID-19. Specifically, patients with vasculitis, other rheumatic and inflammatory diseases or non-Hodgkin lymphoma were affected by higher morbidity and mortality from COVID-19 during the phase of hypogammaglobulinemia and B cell depletion^53–57^. Due to the design of our follow-up study, however, fatal cases of COVID-19 are not represented therein.

IL-6 acts as a major inducer of innate immunity, enhances IgG production by B cells, and has recently emerged as an important biomarker for the early assessment of COVID-19^58^. In our patient cohort, we found a robust relationship between SARS-CoV-2 S-specific IgG concentrations and persistent elevations of IL-6, ferritin, and hepcidin serum concentrations. Given the pleiotropic immunological functions of IL-6, we suggest that these relations are not attributable to statistical associations but rather to mechanistic links. Our data imply that COVID-19 is characterized by a broad and strong innate and adaptive immune response orchestrated by IL-6 and other mediators. Furthermore, our results imply that the magnitude of immune response is interlinked with acute severity and impaired recovery. In this context, we herein demonstrate that persistent inflammation, as reflected by increased IL-6 at follow-up, and elevated serum concentrations of S-specific SARS-CoV-2 IgG are related to delayed pulmonary recovery, including impaired lung function and resolution of COVID-19-related pulmonary CT abnormalities.

Sustained production of IL-6 in severely ill COVID-19 patients may lead to prolonged activation of B cells and stimulate them to produce antibodies that increase both in terms of number and affinity^27^. Furthermore, viral antigens can be retained in secondary lymphoid tissues for several months, allowing affinity maturation to proceed even in the absence of reinfection or vaccination^59^. These antibodies against SARS-CoV-2 can have different specificities and, from an immunological standpoint, can be divided into neutralizing and non-neutralizing idiotypes^30,60–63^. Also, antibodies differ from each other in their immune function and rate of somatic hypermutation^64–67^. The majority of antibodies directed against SARS-CoV-2 are close to the germline configuration. Yet, extensive somatic hypermutation, an indication that antibodies have undergone affinity maturation in germinal centers, is linked to rapid recovery from COVID-19^59^.

In the ongoing COVID-19 pandemic, data from many clinical studies have demonstrated the pleiotropic effects of immune responses against SARS-CoV-2 on patient clinical outcome. Our study adds to this knowledge and describes an independent patient cohort that has been followed up prospectively. Concretely, our data provide evidence that the link between immune activation and disease course is especially relevant for the lungs, which are a primary target for pathogen entry and replication. We used a broad clinical, laboratory, and imaging approach to follow up patients after COVID-19. Our data link the magnitude of the IgG response against the S glycoprotein of SARS-CoV-2 to COVID-19 severity and delayed disease recovery. Although the range of S-specific IgG levels overlapped between fully-recovered patients and those with persistent lung lesions during the observational study period, our results imply that implementing the measurement of SARS-CoV-2 specific IgG levels in the early covalescent phase of COVID-19 in clinical settings may help preselect for patients who might benefit from closer subsequent follow-up care. Thus, the combined use of laboratory and pulmonary function testing may help to avoid unnecessary CT in patients with otherwise unremarkable results. Therefore, limiting the use of CT to individual patients whose laboratory results and pulmonary functions remain altered, or to patients with a discrepancy between poor clinical recovery and unremarkable laboratory and lung function testing, may be a feasible approach that is effective from the perspective of costs, resources, and X-ray exposure. Our study thus provides a framework for systematic and resource sparing follow-up care of larger numbers of COVID-19 patients and deserves validation in independent patient cohorts.

The CovILD study was specifically designed to follow up adult patients after symptomatic COVID-19 and, as such, has several limitations. First, at both ends of the spectrum, asymptomatic and fatal infections with SARS-CoV-2 are not represented in our patient cohort. Rather, approximately three-quarters of the patients in our study were hospitalized for COVID-19, and approximately two-thirds of hospitalized patients required oxygen supplementation without or with subsequent invasive ventilation. Nevertheless, patients with mild to critical disease are equally represented in our cohort. Furthermore, patients with moderate, severe, and critical disease courses are the ones that qualify the most to be screened for potential long-term impairment after COVID-19. Second, patients < 18 years of age met an exclusion criterion and are thus not represented in our study. However, COVID-19 is typically a mild disease in children who therefore may not require extensive work-up by laboratory, pulmonary function, and imaging studies^68^. Third, although our study is multicentric by design, our cohort is rather small. Yet, due to the extensive characterization of the CovILD cohort, the sample size was large enough to identify new robust associations between levels of S-specific IgG and clinical, functional, and CT morphologic findings, resulting in the implementation of a novel pulmonary recovery score.

## Methods

### Patients and study design

The development of interstitial lung disease (ILD) in patients with severe SARS-CoV-2 infection (CovILD) study^45^ is an ongoing prospective, multicenter, observational cohort trial aiming to systematically follow symptomatic patients after COVID-19 (ClinicalTrials.gov number, NCT04416100). The initial diagnosis of COVID-19 was based on typical clinical symptoms and a positive RT-PCR for SARS-CoV-2 obtained from a nasopharyngeal or oropharyngeal swab using the Altona RealStar® SARS-CoV-2 PCR RT-PCR kit 1.0, the Cepheid Xpert® Xpress SARS-CoV-2 test, or the Roche cobas® SARS-CoV-2 test. As a confirmatory test, the Altona or the Roche SARS-CoV-2 RT-PCR was used as needed to definitively establish the diagnosis and guide the ending of isolation. The CovILD study was approved by the local ethics committee at the Medical University Innsbruck (EK Nr: 1103/2020). All research was performed in following relevant guidelines and regulations and is in accordance with the Declaration of Helsinki. Written informed consent was obtained from all study participants and a total of 145 COVID-19 convalescents who previously suffered from mild to critical disease were included in the analysis presented herein as detailed in the Online Supplement.

### Measurement of S-specific IgG levels

IgG antibodies against the S protein of SARS-CoV-2 were quantified with LIAISON® SARS-CoV-2 S1/S2 IgG CLIA (DiaSorin, Italy) and expressed as (AU/ml) using a cut-off of 16.85 AU/ml validated as detailed in the Online Supplement.

### Categorization of clinical severity

We divided the study participants into 4 groups according to clinical disease severity during acute COVID-19 as follows: (1) mild disease: outpatient management without the need for hospitalization; (2) moderate disease: hospitalization without the need for respiratory support; (3) severe disease: hospitalization with the need for oxygen supply; (4) critical disease: ICU treatment with respiratory failure and the need for mechanical ventilation.

### Blood sampling and further analysis

Blood samples were taken via routine peripheral vein puncture and analyzed by standardized ISO-certified procedures. Native or heparinized blood was separated via centrifugation at 300×*g* to collect serum or plasma, respectively, as previously described in detail^69^. Serum samples were frozen at − 30 °C after centrifugation and stored until batch-wise analysis was performed as described in the Online Supplement.

### Analysis of lung involvement with computed tomography

60 days after the diagnosis of COVID-19, we evaluated all study participants with a low-dose (100 kVp tube potential) computed tomography (CT) scan of the chest without the use of an iodine contrast agent as described^44^. CT was acquired on a 128 slice multidetector CT hardware with a 38.4 × 0.6 mm collimation and spiral pitch factor of 1.1 (SOMATOM Definition Flash, Siemens Healthineers, Erlangen, Germany). CT images were evaluated as detailed in the Online Supplement.

### Statistical analysis

Statistical analyses were performed with statistical analysis software package (IBM SPSS Statistics version 26.0, IBM, USA) and R programming suite version 4.0.3. Descriptive statistics included tests for homoscedasticity and data distribution (Levene test, Kolmogorov–Smirnov test, Shapiro–Wilk test, and density blot/histogram analysis). According to explorative data analysis, we used the following tests: Mann–Whitney *U* test, Kruskal–Wallis and Friedman’s test for group comparisons of continuous data, Fisher’s exact test, or Chi-Square test for binary and categorical data. Multiple testing was adjusted by the Sidak formula as appropriate. For the development of a pulmonary recovery score, we used logistic regression as detailed in the Online Supplement.

## Supplementary Information

Supplementary Information.

## Data Availability

The datasets generated during and/or analysed during the current study are available from the corresponding authors upon reasonable request.

## References

[1] Wiersinga WJ, Rhodes A, Cheng AC, Peacock SJ, Prescott HC (2020). Pathophysiology, transmission, diagnosis, and treatment of coronavirus disease 2019 (COVID-19): A review. JAMA.

[2] Streeck H (2020). Infection fatality rate of SARS-CoV2 in a super-spreading event in Germany. Nat. Commun..

[3] Buitrago-Garcia D (2020). Occurrence and transmission potential of asymptomatic and presymptomatic SARS-CoV-2 infections: A living systematic review and meta-analysis. PLoS Med..

[4] Tay MZ, Poh CM, Renia L, MacAry PA, Ng LFP (2020). The trinity of COVID-19: Immunity, inflammation and intervention. Nat. Rev. Immunol..

[5] Nishiga M, Wang DW, Han Y, Lewis DB, Wu JC (2020). COVID-19 and cardiovascular disease: From basic mechanisms to clinical perspectives. Nat. Rev. Cardiol..

[6] Duggal NK, Emerman M (2012). Evolutionary conflicts between viruses and restriction factors shape immunity. Nat. Rev. Immunol..

[7] Merad M, Martin JC (2020). Pathological inflammation in patients with COVID-19: A key role for monocytes and macrophages. Nat. Rev. Immunol..

[8] Cao X (2020). COVID-19: Immunopathology and its implications for therapy. Nat. Rev. Immunol..

[9] Sariol A, Perlman S (2020). Lessons for COVID-19 immunity from other coronavirus infections. Immunity.

[10] Zhao J (2020). Antibody responses to SARS-CoV-2 in patients with novel coronavirus disease 2019. Clin. Infect. Dis..

[11] Braun J (2020). SARS-CoV-2-reactive T cells in healthy donors and patients with COVID-19. Nature.

[12] Meckiff BJ (2020). Imbalance of regulatory and cytotoxic SARS-CoV-2-reactive CD4(+) T cells in COVID-19. Cell.

[13] Vashist SK (2020). In vitro diagnostic assays for COVID-19: Recent advances and emerging trends. Diagnostics.

[14] Espejo AP (2020). Review of current advances in serologic testing for COVID-19. Am. J. Clin. Pathol..

[15] Irsara C (2021). Clinical validation of the Siemens quantitative SARS-CoV-2 spike IgG assay (sCOVG) reveals improved sensitivity and a good correlation with virus neutralization titers. Clin. Chem. Lab. Med..

[16] Irsara C (2020). Evaluation of four commercial, fully automated SARS-CoV-2 antibody tests suggests a revision of the Siemens SARS-CoV-2 IgG assay. Clin. Chem. Lab. Med..

[17] Batra M (2021). Role of IgG against N-protein of SARS-CoV2 in COVID19 clinical outcomes. Sci. Rep..

[18] Ma H (2020). Serum IgA, IgM, and IgG responses in COVID-19. Cell. Mol. Immunol..

[19] Klingler J (2020). Role of IgM and IgA antibodies in the neutralization of SARS-CoV-2. J. Infect. Dis..

[20] Yu HQ (2020). Distinct features of SARS-CoV-2-specific IgA response in COVID-19 patients. Eur. Respir. J..

[21] Iyer AS (2020). Persistence and decay of human antibody responses to the receptor binding domain of SARS-CoV-2 spike protein in COVID-19 patients. Sci. Immunol..

[22] Sterlin D (2020). IgA dominates the early neutralizing antibody response to SARS-CoV-2. Sci. Transl. Med..

[23] Wang Z (2020). Enhanced SARS-CoV-2 neutralization by dimeric IgA. Sci. Transl. Med..

[24] Orth-Holler D, Eigentler A, Stiasny K, Weseslindtner L, Most J (2020). Kinetics of SARS-CoV-2 specific antibodies (IgM, IgA, IgG) in non-hospitalized patients four months following infection. J. Infect..

[25] Isho B (2020). Persistence of serum and saliva antibody responses to SARS-CoV-2 spike antigens in COVID-19 patients. Sci. Immunol..

[26] Ripperger TJ (2020). Orthogonal SARS-CoV-2 serological assays enable surveillance of low-prevalence communities and reveal durable humoral immunity. Immunity.

[27] Pichler D (2021). Marked increase in avidity of severe acute respiratory syndrome coronavirus-2 (SARS-CoV-2) antibodies 7–8 months after infection is not diminished in old age. J. Infect. Dis..

[28] Yao L (2020). Persistence of antibody and cellular immune responses in COVID-19 patients over nine months after infection. J. Infect. Dis..

[29] Figueiredo-Campos P (2020). Seroprevalence of anti-SARS-CoV-2 antibodies in COVID-19 patients and healthy volunteers up to 6 months post disease onset. Eur. J. Immunol..

[30] Gaebler C (2020). Evolution of antibody immunity to SARS-CoV-2. Nature.

[31] Zohar T (2020). Compromised humoral functional evolution tracks with SARS-CoV-2 mortality. Cell.

[32] De Giorgi V (2020). Naturally acquired SARS-CoV-2 immunity persists for up to 11 months following infection. J. Infect. Dis..

[33] Lynch KL (2020). Magnitude and kinetics of anti-severe acute respiratory syndrome coronavirus 2 antibody responses and their relationship to disease severity. Clin. Infect. Dis..

[34] Haddad NS (2020). Elevated SARS-CoV-2 antibodies distinguish severe disease in early COVID-19 infection. BioRxiv.

[35] Phipps WS (2020). SARS-CoV-2 antibody responses do not predict covid-19 disease severity. Am. J. Clin. Pathol..

[36] Ng KW (2020). Preexisting and de novo humoral immunity to SARS-CoV-2 in humans. Science.

[37] Weisberg SP (2020). Distinct antibody responses to SARS-CoV-2 in children and adults across the COVID-19 clinical spectrum. Nat. Immunol..

[38] Al-Samkari H (2020). COVID-19 and coagulation: Bleeding and thrombotic manifestations of SARS-CoV-2 infection. Blood.

[39] Li H (2020). SARS-CoV-2 and viral sepsis: Observations and hypotheses. Lancet.

[40] Zhang Y (2020). Coagulopathy and antiphospholipid antibodies in patients with covid-19. N. Engl. J. Med..

[41] Abers MS (2020). An immune-based biomarker signature is associated with mortality in COVID-19 patients. JCI Insight.

[42] Broman N (2020). IL-6 and other biomarkers as predictors of severity in COVID-19. Ann. Med..

[43] Liu T (2020). The role of interleukin-6 in monitoring severe case of coronavirus disease 2019. EMBO Mol. Med..

[44] Sonnweber T (2020). Persisting alterations of iron homeostasis in COVID-19 are associated with non-resolving lung pathologies and poor patients' performance: a prospective observational cohort study. Respir. Res..

[45] Sonnweber T (2020). Cardiopulmonary recovery after COVID-19 - an observational prospective multi-center trial. Eur. Respir. J..

[46] Fajgenbaum DC, June CH (2020). Cytokine storm. N. Engl. J. Med..

[47] Mangalmurti N, Hunter CA (2020). Cytokine storms: Understanding COVID-19. Immunity.

[48] Kash JC (2006). Genomic analysis of increased host immune and cell death responses induced by 1918 influenza virus. Nature.

[49] Kobasa D (2007). Aberrant innate immune response in lethal infection of macaques with the 1918 influenza virus. Nature.

[50] Moore JB, June CH (2020). Cytokine release syndrome in severe COVID-19. Science.

[51] Leisman DE (2020). Cytokine elevation in severe and critical COVID-19: A rapid systematic review, meta-analysis, and comparison with other inflammatory syndromes. Lancet Respir. Med..

[52] Remy KE (2020). Severe immunosuppression and not a cytokine storm characterizes COVID-19 infections. JCI Insight.

[53] Tepasse PR (2020). Persisting SARS-CoV-2 viraemia after rituximab therapy: Two cases with fatal outcome and a review of the literature. Br. J. Haematol..

[54] Loarce-Martos J (2020). High rates of severe disease and death due to SARS-CoV-2 infection in rheumatic disease patients treated with rituximab: A descriptive study. Rheumatol. Int..

[55] Duléry R (2021). Prolonged in-hospital stay and higher mortality after Covid-19 among patients with non-Hodgkin lymphoma treated with B-cell depleting immunotherapy. Am. J. Hematol..

[56] Kronbichler A (2021). The COVID-19 pandemic and ANCA-associated vasculitis: Reports from the EUVAS meeting and EUVAS education forum. Autoimmun. Rev..

[57] Calderón-Parra J (2021). Incidence, clinical presentation, relapses and outcome of SARS-CoV-2 infection in patients treated with anti-CD20 monoclonal antibodies. Clin. Infect. Dis..

[58] Hirano T (1986). Complementary DNA for a novel human interleukin (BSF-2) that induces B lymphocytes to produce immunoglobulin. Nature.

[59] Chen Y (2020). Quick COVID-19 healers sustain anti-SARS-CoV-2 antibody production. Cell.

[60] Barnes CO (2020). Structures of human antibodies bound to SARS-CoV-2 spike reveal common epitopes and recurrent features of antibodies. Cell.

[61] Brouwer PJM (2020). Potent neutralizing antibodies from COVID-19 patients define multiple targets of vulnerability. Science.

[62] Prevost J (2020). Cross-sectional evaluation of humoral responses against SARS-CoV-2 spike. Cell. Rep. Med..

[63] Nielsen SCA (2020). Human B cell clonal expansion and convergent antibody responses to SARS-CoV-2. Cell Host Microbe.

[64] Atyeo C (2020). Distinct early serological signatures track with SARS-CoV-2 survival. Immunity.

[65] Bartsch YC (2020). Discrete SARS-CoV-2 antibody titers track with functional humoral stability. Nat. Commun..

[66] Kreer C (2020). Longitudinal isolation of potent near-germline SARS-CoV-2-neutralizing antibodies from COVID-19 patients. Cell.

[67] Robbiani DF (2020). Convergent antibody responses to SARS-CoV-2 in convalescent individuals. Nature.

[68] Gotzinger F (2020). COVID-19 in children and adolescents in Europe: A multinational, multicentre cohort study. Lancet Child. Adolesc. Health.

[69] Sonnweber T (2011). Impact of iron treatment on immune effector function and cellular iron status of circulating monocytes in dialysis patients. Nephrol. Dial Transplant..

