# Supplementary material for: Quantity of IgG response to SARS-CoV-2 spike glycoprotein predicts pulmonary recovery from COVID-19
Source: Sci Rep. 2022 Mar 7;12:3677. doi: 10.1038/s41598-022-07489-6 (PMC8901626; doi:10.1038/s41598-022-07489-6)
Supplement: Supplementary file 1 — Supplementary Information. [file 41598_2022_7489_MOESM1_ESM.pdf]

Quantity of IgG response to SARS-CoV-2 spike glycoprotein predicts pulmonary recovery from COVID-19

Keywords: COVID-19; CT abnormalities; ICU; IgG; Lung function; Recovery; SARS-CoV-2; Spike-glycoprotein.

Manfred Nairz[1,*], Sabina Sahanic[1], Alex Pizzini[1], Anna Böhm[1], Piotr Tymoszuk[1], Anna-Maria Mitterstiller[1], Laura von Raffay[1], Philipp Grubwieser[1], Rosa Bellmann-Weiler[1], Sabine Koppelstätter[1], Andrea Schroll[1], David Haschka[1], Martina Zimmermann[1], Silvia Blunder[1], Kristina Trattnig[1], Helene Naschberger[1], Werner Klotz[1], Igor Theurl[1], Verena Petzer[2], Clemens Gehrer[1], John E. Mindur[3], Anna Luger[4], Christoph Schwabl[4], Gerlig Widmann[4], Günter Weiss[1,5], Judith Löffler-Ragg[1], Ivan Tancevski[1], Thomas Sonnweber[1,*]

[1]Department of Internal Medicine II, Medical University of Innsbruck, Innsbruck, Austria

[2]Department of Internal Medicine V, Medical University of Innsbruck, Innsbruck, Austria

[3]Repertoire Immune Medicines, Cambridge, MA, USA

[4]Department of Radiology, Medical University of Innsbruck, Innsbruck, Austria

[5]Christian Doppler Laboratory for Iron Metabolism and Anemia Research, Medical University of Innsbruck, Innsbruck, Austria

[*]Correspondence: manfred.nairz@i-med.ac.at or thomas.sonnweber@i-med.ac.at

Online Supplement

Supplemental Methods

Patients and study design

Inclusion criteria were: symptomatic COVID-19 confirmed by RT-PCR, hospitalization during acute disease or COVID-19 with symptoms persisting for more than 8 weeks, age ≥18 years, and written informed consent to the study. The definitive diagnosis of COVID-19 was based on typical clinical symptoms and two independent positive RT-PCR results for SARS-CoV-2 obtained from two independent nasopharyngeal or oropharyngeal swabs using the Altona RealStar® SARS-CoV-2 PCR RT-PCR kit 1.0, the Cepheid Xpert® Xpress SARS-CoV-2 test, or the Roche cobas® SARS-CoV-2 test[70]. As a primary diagnostic PCR, we used the Altona RT-PCR, which targets the E and S genes of SARS-CoV-2 with a sensitivity of 0.014 to 0.025 plaque forming units (PFU)/ml, or the Cepheid RT-PCR, which targets the E and N2 genes of SARS-CoV-2 with a sensitivity of 0.005 to 0.020 PFU/ml, or the Roche RT-PCR, which detects SARS-CoV-2 Target 1 and pan-Sarbecovirus Target 2 with a sensitivity of 0.003 to 0.009 fifty-percent tissue culture infective dose ($TCID_{50}$/ml; equalling approximately 0.002 to 0.006 PFU/ml). Subsequently, we used the Altona or the Roche RT-PCR as appropriate to confirm the diagnosis and guide the ending of isolation. Subjects were only classified as COVID-19 patients when both genes were detectable in the primary assay used and when one or two genes were detected in a second pharyngeal sample using an alternate confirmatory RT-PCR test. Exclusion criteria were: lack of a confirmative SARS-CoV-2 RT-PCR, age <18 years, lack or withdrawal of consent, and failure to attend >1 follow-up visit. Study participants were evaluated 60, 100, and 180 days after the diagnosis of COVID-19 by employing clinical examination, a medical history assessment, a structured questionnaire to assess typical COVID-19 symptoms, lung function testing, and the acquisition of blood. Pulmonary function testing included body plethysmography and measurement of the diffusion capacity for carbon monoxide (DLCO). Reference levels for DLCO were calculated from sex, age, and height[71].

Analysis of biomarkers

All biomarkers were measured in EDTA-blood, native serum, or heparinized plasma, respectively, using CE-IVD assays in the Rheumatology and Infectious Diseases Laboratory (RILA) and the Central Institute of Clinical and Chemical Laboratory Diagnostics (ZIMCL) of the University Hospital of

Innsbruck. IgG antibodies against the S protein of SARS-CoV-2 were quantified with LIAISON® SARS-CoV-2 S1/S2 IgG CLIA (DiaSorin, Italy) and expressed as arbitrary units per ml of native serum (AU/ml). According to the manufacturer's specifications, the quantitative test results are classified into 3 qualitative categories, i.e. negative (< 12 AU/ml), border-line (12-15 AU/ml), and positive (>15 AU/ml) for SARS-CoV-2 S1/S2 IgG. Before use, the CLIA was validated for diagnostic purposes according to EN ISO15189:2012 demands using serum samples stored at -30°C of 4 independent cohorts of subjects (i.e. 26 patients with symptomatic COVID-19 collected in March and April 2020, 66 healthy volunteers, 50 patients with respiratory infections, and 33 patients with rheumatologic disorders collected before 2019). The validation yielded a cut-off between negative and positive (16.85 AU/ml; sensitivity 100%, 95% CI: 86.77 – 100.0%; specificity 98.66%, 95% CI: 95.24 – 99.84%) as determined by receiver operating curve (ROC) analysis. This high specificity determined in our validation is in accordance with other studies and renders the CLIA highly suitable for cohorts with high prevalence[72,73].

Analysis of lung involvement with computer tomography

CT images were evaluated for the presence of ground-glass opacities (GGO), consolidations, bronchial dilation, and reticulations as defined by the glossary of terms of the Fleischner society[74]. The severity of pulmonary CT abnormalities was graded for every lobe using the following severity score: 0-none, 1-minimal (subtle GGO, very few findings), 2-low (several GGO, subtle reticulation), 3-moderate (multiple GGO, reticulation, small consolidation), 4-marked (extensive GGO, consolidation, reticulation with distortion), and 5-massive (massive findings, parenchymal destructions). The maximum score was 25 (i.e. maximum score 5 per lobe).

Development of the pulmonary recovery score

For the development of a pulmonary recovery score, correlation of clinical, demographic, and biochemical risk factors for persisting pulmonary abnormalities (CT abnormalities and CT severity score >5) at the 180-day follow-up were investigated with univariate and multivariate logistic regression. The significance of model estimates was tested with Wald's Z test. For the multivariate tests, the significance of model components was additionally corroborated with LRT (likelihood ratio test, Chi^2 test for Δ deviance $\neq 0$). The final recovery score was calculated with a formula based on linear estimates of the multivariate logistic model for the risk of CT abnormalities. Prediction accuracy of the recovery score was verified by receiver operator characteristic analysis (ROC, R

packages plotROC, and optimalCutpoints) [75]. Optimal cutpoints in ROC were determined by the Youden method.

Supplemental References

70 Nairz, M. *et al*. Overcoming limitations in the availability of swabs systems used for SARS-CoV-2 laboratory diagnostics. *Sci Rep* **11**, 2261. doi:10.1038/s41598-021-81782-8 (2021).

71 Cotes, J. E. *et al*. Standardization of the measurement of transfer factor (diffusing capacity). *Eur Respir J* **6**, 41-52. doi:10.1183/09041950.041s1693 (1993).

72 Perkmann, T. *et al*. Side-by-Side Comparison of Three Fully Automated SARS-CoV-2 Antibody Assays with a Focus on Specificity. *Clin Chem* **66**, 1405-1413. doi: 10.1093/clinchem/hvaa198 (2020).

73 Bird, P. W. *et al*. Retrospective serosurveillance for anti-SARS-CoV-2 immunoglobulin during a time of low prevalence: A cautionary tale. *J Infect* **83**, 119-145. doi: 10.1016/j.jinf.2021.03.007 (2021).

74 Hansell, D. M. *et al*. Fleischner Society: glossary of terms for thoracic imaging. *Radiology* **246**, 697-722. doi:10.1148/radiol.2462070712 (2008).

75 Sachs, M. C. plotROC: A Tool for Plotting ROC Curves. *J Stat Softw* **79**. doi:10.18637/jss.v079.c02 (2017).
